# Inside the Shark Nursery: The Evolution of Live Birth in Cartilaginous Fish

**DOI:** 10.1093/gbe/evad037

**Published:** 2023-03-15

**Authors:** Casey McGrath

*A new study in Genome Biology and Evolution reveals that egg yolk proteins may have been co-opted to provide maternal nutrition in live-bearing sharks and their relatives.*

**Fig. 1.**
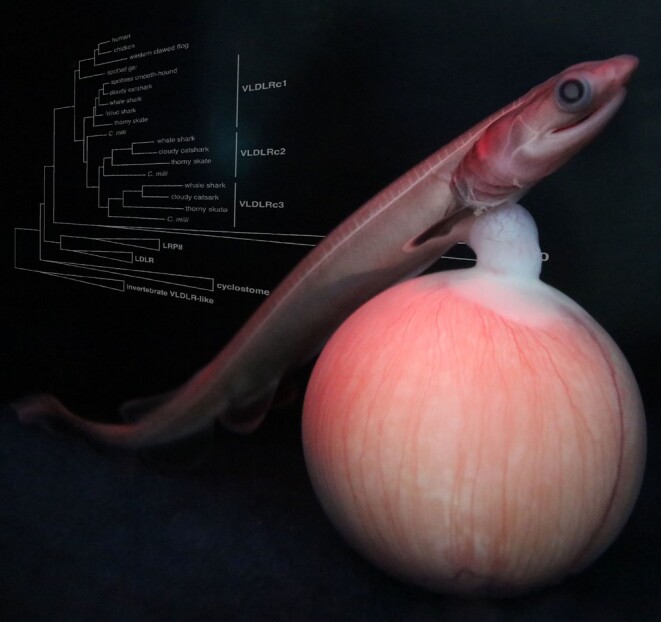
A developing embryo of the frilled shark, which has a unique mode of live-bearing and is thought to exhibit a long gestation time of no less than three years. Photograph by Frilled Shark Research Project.

While giving birth to live young is a trait that most people associate with mammals, this reproductive mode—also known as viviparity—has evolved over 150 separate times among vertebrates, including over 100 independent origins in reptiles, 13 in bony fishes, 9 in cartilaginous fishes, 8 in amphibians, and 1 in mammals. Hence, understanding the evolution of this reproductive mode requires the study of viviparity in multiple lineages. Among cartilaginous fishes—a group including sharks, skates, and rays—up to 70% of species give birth to live young (fig. 1); however, viviparity in these animals remains poorly understood due to their elusiveness, low fecundity, and large and repetitive genomes. In a recent article published in *Genome Biology and Evolution*, a team of researchers led by Shigehiro Kuraku, previously Team Leader at the Laboratory for Phyloinformatics at RIKEN Center for Biosystems Dynamics Research in Japan, set out to address this gap. Their study identified egg yolk proteins that were lost in mammals after the switch to viviparity but retained in viviparous sharks and rays (Ohishi et al. 2023). Their results suggest that these proteins may have evolved a new role in providing nutrition to the developing embryo in cartilaginous fishes.

According to Kuraku, who now works as Professor of Molecular Life History Laboratory at the National Institute of Genetics in Mishima, investigators have long wanted to learn more about the evolution of viviparity in sharks and their relatives. “Reproduction is one of the most fascinating features of cartilaginous fishes because they show a broad spectrum of reproductive modes.” Among viviparous species, this includes a range of mechanisms for providing nutrients to the developing embryo, from relying solely on nutrients present in the embryo's yolk sac, to feeding the embryo unfertilized eggs, secreting nutrients from the uterus (“uterine milk”), or transferring nutrients via a placenta.

To better understand these various mechanisms, the authors searched genomic and transcriptomic data from 12 cartilaginous fishes for homologs of vitellogenin (*VTG*), a major egg yolk protein synthesized in the female liver in egg-laying species. Regardless of their reproductive mode, all cartilaginous fish species had at least two copies of *VTG*, while all copies of *VTG* have been lost from mammals (although the authors did identify a copy in the Tasmanian devil, a marsupial, which was not previously known to harbor a *VTG* gene). Next, the authors searched for homologs of the VTG receptor; while mammals retain a single copy of this receptor, Kuraku and his colleagues identified two ancient tandem duplications giving rise to three copies of the receptor in cartilaginous fishes. The authors note that this finding was unexpected. “We predicted the retention of egg yolk protein genes in the shark genomes because live-bearing sharks rely partly on nutrition supply from the egg yolk,” says Kuraku. “What surprised us the most was that cartilaginous fish including sharks have more copies of the egg yolk protein receptor genes.” This suggested that these proteins may provide a novel function in this viviparous lineage.

To shed light on the functions of VTG and its receptor in these species, the authors compared tissue-by-tissue transcriptome data from one egg-laying shark (the cloudy catshark) and two viviparous sharks. The frilled shark is a viviparous species that provides no maternal nutrients to the developing embryo, while the spotless smooth-hound has a placenta. In the egg-laying cloudy catshark, VTG is primarily expressed in the liver, and its receptors are primarily expressed in the ovary. In contrast, in the two viviparous sharks, VTG was expressed not only in the liver but also in the uterus. Interestingly, the VTG receptor was also expressed in the uterus in these species. This suggests that VTG proteins may not only function as yolk nutrients but may also be transported into the uterus, where they may play a role in providing maternal-based nutrition in some cartilaginous fishes.

As noted by the authors, this intriguing possibility remains to be confirmed through functional studies. They also hope to expand this analysis to a genome-wide survey of factors associated with the various reproductive modes of cartilaginous fishes. Unfortunately, such experiments are difficult in these species given the challenge in obtaining biological samples. Kuraku and his collaborators, however, hope to change this. “This study was enabled by networking among individuals with various types of expertise who recognize the biological potential of cartilaginous fishes,” says Kuraku. “It also led to the launch and development of the Squalomix consortium,” an initiative launched in 2020 to promote genomic and molecular approaches specifically targeting shark and ray species (Nishimura et al. 2022). The consortium aims to make its resources publicly available, including a cell culture technique that may help enable functional assays of molecules (Uno et al. 2020), facilitating future research into the reproductive modes of these elusive and fascinating creatures.
